# Prevalence of child passenger restraint use in Shantou, China from 2012 to 2017

**DOI:** 10.1186/s12889-020-08859-3

**Published:** 2020-05-29

**Authors:** Shuzhen Yan, Kele Ding, Jingzhen Yang, Wanbao Ye, Liping Li

**Affiliations:** 1 Injury Prevention Research Center, Shantou University Medical College, Shantou, Guangdong Province China; 2 Department of Health Sciences, Kent State University, Kent, OH USA; 3 Center for Injury Research and Policy, The Research Institute at Nationwide Children’s Hospital, Columbus, OH USA

**Keywords:** Child passenger safety, Child passenger restraint use, Child restraint system, China

## Abstract

**Background:**

Child passenger safety is an important public health problem in China. This study aimed to examine the prevalence of child passenger restraint use while riding in a car in the city of Shantou in China from 2012 to 2017.

**Methods:**

Three large-scale cross-sectional observational studies were conducted in 2012, 2015 and 2017, respectively. The observation sites included randomly selected hospitals, kindergartens, and primary and secondary schools. The outcome measures included the changes in percentages of seating position (e.g., front vs. rear), whether sitting on lap, and use of child restraint systems (CRS) or seat belts by year and by age group. Descriptive statistics, Chi-square tests and logistic regression were used to address the study aims.

**Results:**

A total of 9858 commuting children aged 17 and younger were observed in passenger cars in Shantou, China during the study. The proportion of children aged 0–5 sitting on adult’s lap decreased from 26.6% in 2012 to 24.6% in 2017, while the proportion of CRS use among the children sitting in the rear row increased among children aged 0–5 (from 0.7% in 2012 to 14.2% in 2017) and children aged 6–11 (from 0.7% in 2012 to 2.4% in 2017). Comparing children aged 0–11 in 2012, children in the same ages were less likely to sit in the front row in 2015 (OR = 0.42, 95%CI = 0.37, 0.48) and in 2017 (OR = 0.27, 95%CI = 0.23, 0.31). Children aged 0–11 were more likely to sit in the rear row with CRS use in 2015 (OR = 8.50, 95%CI = 5.44, 13.28) and in 2017 (OR = 10.95, 95%CI = 7.02, 17.08) comparing with children in the same ages in 2012. As for children aged 12–17, they were more likely to use seat belt in 2017 (OR = 1.40, 95%CI = 1.06, 1.85) compared with those children in 2012.

**Conclusions:**

While child passenger safety behaviors improved from 2012 to 2017 in Shantou, China, more efforts are needed to protect child passengers from injuries.

## Background

The World Health Organization estimates that road crashes result in more than 256,180 deaths in road users every year in China [1]. Motor vehicle crashes remain a leading cause of death among children [2]. Each year, about 100,000 people in China are killed in motor vehicle crashes, and children aged 1–20 years accounted for more than 12% of these deaths [3]. The best solution to protect children from injuries and deaths in car crashes is to use appropriate child restraint systems (CRS). Prior study shows that when CRS is correctly installed and used, it can reduce the risk of death for infants (aged < 1 year) by 71%, and toddlers (aged 1–4 years) by 54% [4]. Additional data suggests restraining children in rear seats instead of front seats could better reduce the risk of serious injury or death in a crash [5, 6].

Mandatory legislation on child safety restraint use is as an effective approach to increase CRS use and reduce injuries among child passengers [7, 8]. According to the Global Status Report on Road Safety 2018, 84 countries have enacted a national child restraint law, with the majority being developed countries [1]. The rates of CRS use in these developed countries are high, including 97% in Austria and 89% in the United States [1]. However, many low-income or developing countries do not have a child restraint law. China, with approximately one-fifth of the total world population, currently has no national child restraint law. The rate of CRS use is less than 1% [1]. Even in cities with local CRS laws (such as Shanghai and Shenzhen), the rate of CRS use among children under the age of 7 is low, ranging from 6.1% in Shanghai, [9] to 22.9% in Shenzhen [10].

Shantou is a coastal city located in the southeast of China with no CRS law. To provide empirical data on current use of CRS in Shantou, our research team conducted three large-scale cross-sectional studies in 2012, [11] 2015, [12] and 2017, [13] respectively. The aim of this study was to describe the prevalence of child passenger restraint use while riding in a car in the Shantou province in China, from 2012 to 2017. Specifically, it described changes in percentages of seating position (e.g., front vs. rear), whether sitting on lap, and use of child restraint systems (CRS) or seat belts by year and by age group. The results of the study will provide empirical data on child passenger restraint use, which could serve as the basis for law makers to develop and enact a child passenger law in Shantou, China.

## Methods

### Study setting and participants

We analyzed the data collected from three cross-sectional studies that were conducted in 2012, 2015, and 2017, respectively. Similar methods were used for the three studies, with each consisting of two components: direct observation and in-person survey. For purpose of this study only data collected from direct observations were analyzed and reported [11, 12].

We conducted direct observations at four types of locations:1) immunization clinics of hospitals, where infants and toddlers aged 3 and younger were the primary population; 2) kindergartens, attended by children aged 3–5; 3) primary schools, attended by children aged 6–11; and 4) middle schools, attended by children aged 12–17. We used multi-stage sampling to select observation sites. We first selected two of seven administrative areas (Longhu region and Jinping region) in Shantou. Then, we randomly selected clinics, kindergartens, primary schools, and middle schools from each region. In 2012, 4 immunization clinics of hospitals, 24 kindergartens, 31 primary schools and 22 middle schools were selected as the study sites. In 2015 and 2017, most study sites were the same as 2012, and included 4 immunization clinics of hospitals, 30 kindergartens, 30 primary schools and 30 middle schools.

### Study procedure

To ensure that the data collected from different observers were reliable and compatible, we trained the observers uniformly. The training included showing the observers different types of CRS, how to conduct observations, how to invite potential participants and the detailed study protocol and steps.

On the day the observations were conducted, two observers were assigned to each of the selected observation sites of hospitals, kindergartens and schools. The observers stood by roads that were close to intersections or had crossroads with red lights, stop signs or traffic lights that made the vehicles stop or slow down, which presented an opportunity for the observers to inspect the vehicle carefully.

Data were collected from each study site for only one time using the established observation instrument [11, 12]. Data collection conducted at the school gate was completed from 16:00–17:30 and at the hospital parking lot was completed from 9:00–12:30 and 14:00–18:00 on weekdays. The reason for choosing this time range was that during this time, children were more likely to commute in cars. If there was more than one child in a car, each child’s information was collected and recorded separately.

The study procedure was approved by the Ethics Committee of the Medical College at Shantou University.

### Study variables

The data collected during the observation included the following dichotomous variables: seat position (front or rear row), sitting on an adult’s lap (yes or no), CRS use (yes or no), and seat belt use (yes or no). In addition, demographic variables including child passengers’ sex and age were collected. Child’s age was collected as a continuous variable initially and was broken down into three categories (0–5, 6–11, and 12–17) according to passenger safety measures and recommendations.

For purpose of the study, CRS use, an outcome of interest, was measured using the following two child passenger safe behaviors: 1) For children aged 0–11, any CRS use including use of a rear-facing safety seat, forward-facing safety, or booster seat while sitting in the rear row, and 2) For children aged 12–17, seat belt use both sitting in the front or rear row. Additionally, we defined the following child passenger risk behaviors: 1) For children of any age, sitting on an adult’s lap, 2) For children aged 0–11, sitting in the front row.

### Statistical analysis

Data were analyzed using SPSS version 23.0 software [14]. Descriptive statistics were used to describe the frequency and percentage of the study variables. Differences in child passenger safe and risk behaviors were compared across years and by age groups using Chi-square Tests. The odds ratios (OR) and 95% confidence interval (CI) of child passenger safe and risk behaviors were estimated by year using Logistic regression with appropriate age sub-groups, adjusting for age and sex. The outcome variables included the two child passenger safe behaviors and two child passenger risk behaviors described above. *P* > 0.05 was defined as significance level.

## Results

During the study period, a total of 9858 commuting children aged 17 and younger were observed in passenger cars in Shantou, China. Table 1 showed the demographic information of the study participants by years. For the restraint use of child passengers, an increased percentage was observed from 2012 to 2017 in any CRS use and in seat belt use. The percentage of CRS use ranged from 0.7 to 6.2%, and the percentage of seatbelt use ranged from 8.9 to 12.7%. Additionally, we also observed decreased percentages of children sitting on an adult’s lap from 16.8% in 2012 to 10.5% in 2017.

**Table 1:** Demographics of restrain use of child passengers by study year (n/%)

Items	2012	2015	2017	Total
**Total**	3333	3464	3061	9858
**Child gender**				
Male	1691 (50.7)	1705 (49.2)	1617 (52.8)	5013 (50.9)
Female	1439 (43.2)	1582 (45.7)	1302 (42.6)	4323 (43.8)
Unknown	203 (6.1)	177 (5.1)	142 (4.6)	522 (5.3)
**Child age (years)**				
0–5	2045 (61.4)	1807 (52.2)	1226 (40.1)	5078 (51.5)
6–11	817 (24.5)	1043 (30.1)	1217 (39.8)	3077 (31.2)
12–17	471 (14.1)	614 (17.7)	618 (20.1)	1703 (17.3)
**Seat position**				
Front row	1011 (31.8)	630 (18.2)	426 (13.9)	2067 (21.0)
Rear row	2322 (68.2)	2834 (81.8)	2635 (86.1)	7791 (79.0)
**Sit on an adult’s lap**				
Yes	555 (16.8)	319 (9.2)	321 (10.5)	1195 (12.2)
No	2778 (83.2)	3145 (90.8)	2740 (89.5)	8639 (87.8)
**Restraint type**				
CRS	22 (0.7)	189 (5.5)	191 (6.2)	402 (4.1)
Safety belt	297 (8.9)	316 (9.1)	390 (12.7)	1003 (10.2)
Unrestrained	3014 (90.4)	2959 (85.4)	2480 (81.0)	8453 (85.7)

Note: Each percent in this table was column percent

The American Academy of Pediatrics recommends 5 evidence-based best practices in the choice of a child restraint system in passenger vehicles from birth through adolescence [15]. Table 2 provided a summary of observed risk and safety behaviors in percentages across the years classified based on the recommendations. For children aged 0–5, we observed the proportion of sitting on an adult’s lap decreased from 26.6% in 2012 to 24.6% in 2017. The proportion of sitting in the front row decreased from 28.3% in 2012 to 7.0% in 2017. Meanwhile, the proportion of CRS use among the children sitting in the rear row increased from 0.7% in 2012 to 14.2% in 2017. For children aged 6–11, we observed the proportion of sitting in the front row decreased from 32.7% in 2012 to 13.2% in 2017 while the proportion of CRS use among the children sitting in the rear row increased from 0.7% in 2012 to 2.4% in 2017. For children aged 12–17, we observed the proportion declined from 2012 to 2017 in sitting in the front row while the proportion of sitting in the rear row increased from 64.8% in 2012 to 71.0% in 2017. However, the proportion of children aged 12–17 sitting in the rear row with seatbelt use did not increase.

**Table 2:** Prevalence of risk and protective behaviors by age groups by study year

	2012			2015			2017			Total			
	n	%	95%CI	n	%	95%CI	n	%	95%CI	n	%	95%CI	***P-value***
**Total**	3333			3464			3061			9858			
**Age 0-5 group**	2045			1807			1226			5078			
Sit on an adult's lap^a^	543	26.6	24.7-28.5	289	16.0	14.3-17.7	302	24.6	22.2-27.0	1134	22.3	21.2-23.4	<0.001
Front row^a^	578	28.3	26.3-30.3	227	12.6	11.1-14.1	86	7.0	5.6-8.4	891	17.5	16.5-18.5	
Front row without CRS	571	98.8	98.3-99.3	226	99.6	99.3-99.9	83	96.5	95.5-97.5	880	98.8	98.5-99.1	0.126
Front row with CRS	7	1.2	0.7-1.7	1	0.4	0.1-0.7	3	3.5	2.5-4.5	11	1.2	0.9-1.5	
Rear row	1467	71.7	69.7-73.7	1580	87.4	85.9-88.9	1140	93.0	91.6-94.4	4187	82.5	81.5-83.5	
Rear row without CRS	1457	99.3	98.9-99.7	1419	89.8	88.4-91.2	978	85.8	83.8-87.8	3854	92.0	91.3-92.7	<0.001
Rear row with CRS^b^	10	0.7	0.3-1.1	161	10.2	8.8-11.6	162	14.2	12.2-16.2	333	8.0	7.3-8.7	
**Age 6-11 group**	817			1043			1217			3077			
Sit on an adult's lap^a^	9	1.1	0.4-1.8	27	2.6	1.6-3.6	19	1.6	0.9-2.3	55	1.8	1.3-2.3	0.042
Front row^a^	267	32.7	29.5-35.9	199	19.1	16.7-21.5	161	13.2	11.3-15.1	627	20.4	19.0-21.8	
Front row without CRS	266	96.6	99.2-100	196	98.5	97.8-99.2	160	99.4	99.0-99.8	622	99.2	98.9-99.5	N/A
Front row with CRS	1	0.4	0-0.8	3	1.5	0.8-2.2	1	0.6	0.2-1.0	5	0.8	0.5-1.1	
Rear row	550	67.3	64.1-70.5	844	80.9	78.5-83.3	1056	86.8	84.9-88.7	2450	79.6	78.2-81	
Rear row without CRS	546	99.3	98.7-99.9	833	98.7	98.0-99.4	1031	97.6	96.7-98.5	2410	98.4	98-98.8	0.031
Rear row with CRS^b^	4	0.7	0.1-1.3	11	1.3	0.6-2.0	25	2.4	1.5-3.3	40	1.6	1.2-2.0	
**Age 12-17 group**	471			614			618			1703			
Sit on an adult's lap^a^	3	0.6	0.1-1.3	3	0.5	0.1-1.1	0	0.0	0-0	6	0.4	0.1-0.7	N/A
Front row^a^	166	35.2	30.9-39.5	204	33.2	29.5-36.9	179	29.0	25.4-32.6	549	32.2	30-34.4	
Front row without seat belt	97	58.4	53.9-62.9	114	55.9	52-59.8	32	17.9	14.9-20.9	243	44.3	41.9-46.7	<0.001
Front row with seat belt	69	41.6	37.1-46.1	90	44.1	40.2-48.0	147	82.1	79.1-85.1	306	55.7	53.3-58.1	
Rear row	305	64.8	60.5-69.1	410	66.8	63.1-70.5	439	71.0	67.4-74.6	1154	67.8	65.6-70.0	
Rear row without seat belt	269	88.2	85.3-91.1	386	94.1	92.2-96	409	93.2	91.2-95.2	1064	92.2	90.9-93.5	0.01
Rear row with seat belt ^b^	36	11.8	8.9-14.7	24	5.9	4.0-7.8	30	6.8	4.8-8.8	90	7.8	6.5-9.1	

Note: Denominators by age groups by year were shown in Table 1

*N/A* Not available

^**a**^ risk behaviors

^b^ safe behavior

Table 3 compared age groups and their odds of engaging in each of the passenger safety behaviors. Children aged 0–17 were less likely to sit on an adult’s lap in 2015 (OR = 0.51, 95%CI = 0.42, 0.59) and in 2017 (OR = 0.59, 95%CI = 0.51, 0.68) compared with children in the same ages in 2012. Comparing children aged 0–11 in 2012, children in the same ages were less likely to sit in the front row in 2015 (OR = 0.42, 95%CI = 0.37, 0.48) and in 2017 (OR = 0.27, 95%CI = 0.23, 0.31). Children aged 0–11 were more likely to sit in the rear row with CRS use in 2015 (OR = 8.50, 95%CI = 5.44, 13.28) and in 2017 (OR = 10.95, 95%CI = 7.02, 17.08) comparing with children in the same ages in 2012. As for children aged 12–17, they were more likely to use seat belt in 2017 (OR = 1.40, 95%CI = 1.06, 1.85) compared with those children in 2012 but no statistically significant difference in 2015 (*P*>0.05).

**Table 3:** Odds ratios of child passenger safe and risk behaviors overtime by study years

Items	2012	2015	2017
**Sit on an adult’s lap**^**a**^			
Age 0–17 group	Reference	0.51 (0.42,0.59)^**^	0.59 (0.51,0.68) ^**^
**Sit in the front row**			
Age 0–11 group	Reference	0.42 (0.37,0.48)^**^	0.27 (0.23,0.31)^**^
**Sit in the rear row, CRS use**			
Age 0–11 group	Reference	8.50 (5.44,13.28)^**^	10.95 (7.02,17.08)^**^
**Seat belt use**			
Age 12–17 group	Reference	0.80 (0.59,1.07)	1.40 (1.06,1.85)^*^

OR value was obtained by conducting logistic regression with selected age sub-group as listed. OR was adjusted for child’s gender and age. *P<0.05. ** *P*<0.001

## Discussion

This study described the prevalence of safe restraint use among child passengers in Shantou, China through three observation studies conducted in 2012, 2015, and 2017. The results showed that about one-fourth (22.3%) of children aged 0–5 were sitting on an adult’s lap without using any safe restraint. This is likely due to some Chinese parents’ wrong belief that holding their younger children on their laps is the best way to protect them from injury in the car, [11, 16] and/or a lack of awareness that an appropriate child car restraint is highly effective in reducing the risk of death or injury for child car passengers in a crash [15, 17, 18]. The American Academy of Pediatrics (AAP) has provided 5 evidence-based recommendations for best practices in the choice of a CRS to optimize safety in passenger vehicles for children according to their height, weight and age [15]. The first recommendation is that rear-facing safety seats should be used for most infants up to 2 years of age. The second is that forward-facing safety seats should be used for most children through 4 years of age. Although findings showed that children aged 0–5 were less likely to sit on an adult’s lap in 2015 and in 2017, continued efforts are needed to educate and encourage Chinese parents to use CRS based on their child’s height, weight and age.

Our findings show that one-fifth (20.4%) of children aged 6–11 were sitting in the front seat, and only 1.6% children were using a CRS among the children sitting in the rear row. To a certain degree, this reflects the fact CRS is not often used for children while traveling in a car [11, 16]. Based on the American Academy of Pediatrics recommendation, a fifth evidence-based recommendation is for all children younger than 13 years to ride in the rear seats of vehicles [15]. Existing evidence also shows that children sitting in the rear of the car are safer than children who sit in the front [6, 19]. Therefore, children aged 6–11 should ride in the rear row with an appropriate CRS for effective protection until they have outgrown booster seats. When children become age 12 or older, they should use lap and shoulder seat belts for optimal protection regardless of their position in the front or rear row. However, our findings showed that 44.3% of children aged 12–17 did not use a seat belt when they were sitting in the front, and even fewer children (7.8%) used a seat belt when they were sitting in the rear row. Our findings on seatbelt use among children aged 12–17 while travelling in a car are still quite low in 2017 suggest additional efforts need to be made to develop and implement effective interventions targeting increasing seatbelt use among children while travelling in a car.

From 2012 to 2017, the proportion of children aged 0–5 who were sitting on an adult’s lap decreased by year; similarly, children aged 0–11 sitting in the front row decreased by year. Meanwhile, the proportion of CRS use among children aged 0–5 increased by year. This progress is very encouraging, but not surprising, because our research team conducted four educational interventions targeting child passenger safety in 2013, [20] 2015, [21] 2016, [22] and 2017, [13] respectively. Through our educational programs, more people in our city, Shantou, may become aware of the importance of using safety restraint for child passengers. All of these factors may have positively impacted the awareness of safety measures for child passengers. Despite the increased awareness, the rate of child restraint use was still very low, with only 7.8% of children using CRS in Shantou [23]. Risk behaviors are still quite prevalent and remain a serious safety issue. Additional efforts are urgently needed to combat these risk behaviors.

Documenting the prevalence of child passenger safety practices could be an important indicator for the effectiveness of public information campaigns and child passenger safety programs. Based on our findings, we could make one recommendation for preventing road traffic injuries and deaths among Chinese children. That is to continue educational efforts and take additional measures to increase knowledge of road safety, reduce traffic risk behaviors, and increase safe behaviors to protect child passengers. These educational interventions may use strategies that have had promising results in high-income countries [23, 24] and include community based education CRS programs, maternity hospital CRS loan programs, voucher programs to encourage subsidized purchase of CRS, and CRS checking programs that verify correct fit with the child.

This study has several limitations. First, data on the child’s height and weight were not collected. Given that belt-positioning booster seats should be used for most children through at least 8 years of age; and lap and shoulder seat belts for all who have outgrown booster seats, we could not analyze whether children aged 6–11 were meeting the recommendations. Second, our observers were not able to determine which type of CRS was used in all cars, if it was exactly appropriate for the child’s age, height or weight, or if it was properly installed or fastened correctly. Third, we only tried to determine the prevalence of using CRS in private cars. Other vehicles such as taxis, vans, buses, and pick-up trucks were not included. Finally, the prevalence of the CRS use in Shantou reported in this study was based on the data collected during weekdays, which may not reflect the CRS use during weekends, and our findings may not be generalizable to other cities or provinces in China.

## Conclusion

In general, child passenger safety behaviors improved from 2012 to 2017 in Shantou, China. Specifically, the proportion of children aged 0–5 who used CRS and the proportion of children aged 12–17 who used a seat belt increased by year. Additional efforts are needed to educate the public about the benefits and importance of CRS use and to reduce risk behaviors in order to protect child passengers while travelling in a car.

## Data Availability

Not applicable.

## Abbreviations

CRS: child restraint systems
OR: odds ratios
CI: confidence interval
AAP: American Academy of Pediatrics
